# Fentanyl versus Methadone in Management of Withdrawal Syndrome in Opioid Addicted Patients; a Pilot Clinical Trial 

**DOI:** 10.22037/aaem.v9i1.1384

**Published:** 2021-09-13

**Authors:** Baharak Najafi, Shahin Shadnia, Hossein Hassanian-Moghaddam, Amir Heydarian, Arezou Mahdavinejad, Nasim Zamani

**Affiliations:** 1 Department of Clinical Toxicology, Loghman Hakim Hospital, Shahid Beheshti University of Medical Sciences, Tehran, Iran.; 2 Social Determinants of Health Research Center, Shahid Beheshti University of Medical Sciences, Tehran, Iran.; 3 Department of Emergency Medicine, Loghman Hakim Hiospital, Shahid Beheshti University of Medical Sciences, Tehran, Iran.; 4 Toxicological Research Center, Shahid Beheshti University of Medical Sciences, Tehran, Iran.

**Keywords:** Methadone, Fentanyl, Substance withdrawal syndrome, Drug therapy, Intensive care units

## Abstract

**Introduction:**

The most effective treatment for withdrawal syndrome in Opioid-dependent patients admitted to intensive care units (ICUs) remains unknown. This study aimed to compare fentanyl and methadone in this regard.

**Methods:**

This prospective, single-blinded, controlled pilot study was conducted on opioid-dependent intubated patients admitted to the toxicology ICU of Loghman Hakim Hospital, Tehran, Iran, between August 2019 and August 2020. Patients were alternately assigned to either fentanyl or methadone group after the initiation of their withdrawal syndrome. Duration and alleviation of the withdrawal signs and symptoms, ICU and hospital stay, development of complications, development of later signs/symptoms of withdrawal syndrome, and need for further administration of sedatives to treat agitation were then compared between these two groups.

**Results:**

Median age of the patients was 42 [interquartile range (IQR): 26, 56]. The two groups were similar in terms of the patients’ age (p = 0.92), sex (p = 0.632), primary Simplified Acute Physiology Score (SAPS) II (p = 0.861), and Clinical Opiate Withdrawal Score (COWS) before (p = 0.537) and 120 minutes after treatment (p = 0.136) with either methadone or fentanyl. The duration of intubation (p = 0.120), and ICU stay (p = 0.572), were also similar between the two groups. The only factor that was significantly different between the two groups was the time needed for alleviation of the withdrawal signs and symptoms after the administration of the medication, which was significantly shorter in the methadone group (30 vs. 120 minutes, p = 0.007).

**Conclusion:**

It seems that methadone treats the withdrawal signs and symptoms faster in dependent patients. However, these drugs are similarly powerful in controlling the withdrawal signs in these patients.

## 1. Introduction:

Substance use disorder is a common problem although its prevalence in the inpatient setting is not well-defined. In 2012, it was estimated that 11% of adult hospitalizations involved substance use disorders; however, this is probably an underestimation, considering the high frequency of underdiagnosis of substance use disorders (1). Withdrawal syndrome is a major problem in opioid-dependent patients when they are admitted and stay at the hospital for a relatively long period of time, such as when they are admitted to intensive care units (ICUs) for any reason. In toxicology ICUs, the situation is even more complicated as the dependent patient has already overdosed on a substance or medication making the selection of the best drug/medication for treatment of withdrawal a challenge.

Different medications have been proposed as substitutes for opioids but it is generally accepted that the best drug/medication for treatment of withdrawal syndrome is the one the patient is dependent on (2). However, management of critically ill or injured patients who use illicit substances is complicated due to both the intoxicating and the withdrawal effects of those substances. On the other hand, some medications (including tramadol, oral methadone, opium tincture, etc.) are not available in hospital settings and this may complicate the process of treatment of withdrawal syndrome in dependent patients. The complex physiologic responses of these patients as well as their loss of consciousness during the withdrawal may further complicate their management (3). In addition, poor management of withdrawal syndrome may cause severe agitation, increase the need for sedation, postpone extubation, and increase the hospital stay. 

Thus, finding the best medication to control withdrawal syndrome in dependent patients admitted to the ICU is a major challenge, a fact that has been overlooked in the existing literature. The aim of the current study was to compare fentanyl, as a routinely administered drug in ICUs to control both withdrawal and pain, with methadone, as a widely accepted medication to treat withdrawal syndrome in hospital-admitted opioid-dependent patients.

## 2. Methods:

***2.1. Study design and setting***

In a prospective, single-blinded, controlled pilot study, opioid-dependent intubated patients admitted to toxicology ICU of Loghman Hakim Hospital between August 2019 and August 2020 were alternately assigned to either fentanyl or methadone groups. The study was approved by our local Ethics Committee in Shahid Beheshti University of Medical Sciences under the code IR.SBMU.RETECH.REC.1398.457 and registered on Iranian registry of clinical trials (IRCT: IRCT20150110020624N2). Written consents were obtained from the patients’ relatives on ICU admission. 

***2.2. Participants***

Addicted patients who were intubated and their signs and symptoms of withdrawal initiated during ICU stay were included. Any severity of withdrawal symptoms was considered as the inclusion criteria (mild, moderate, and severe withdrawal symptoms). Patients with other dependencies (such as dependency to benzodiazepines) were excluded. 

***2.3. Interventions***

The patients were then alternately assigned to either methadone or fentanyl groups. Only the patients were blinded to the type of treatment they were given as they were unconscious and intubated. In the methadone group, they were put on subcutaneous methadone (Faran Shimi Company, Iran) with the initial dose of 10 mg every 12 hours (20 mg/day) in the beginning. The dose was subsequently adjusted based on the patients’ withdrawal signs and symptoms. In the fentanyl group, the patients were put on intravenous administration of fentanyl (Faran Shimi Company, Iran) with the initial dose of 50-100 μg/hour, which was subsequently adjusted based on the patients’ response. The mean dose of fentanyl was considered for analysis. For instance, if a patient received fentanyl with the dose 50-100 μg/hour, a mean dose of 75 μg/hour was calculated and considered for analysis. Half an hour and two hours later the patients were re-visited and their Clinical Opiate Withdrawal Score (COWS) was re-calculated. Clonidine was initiated and continued in all patients with the initial dose of 0.1 mg every eight hours (0.3 mg/day) and adjusted to a maximum daily dose of 1.2 mg. 

***2.4. Data gathering***

Demographic data and severity of symptoms as well as outcomes were recorded for all cases using a predesigned checklist. N.Z was responsible for data gathering.

***2.5. Outcomes***

The primary outcome evaluated was alleviation of the withdrawal signs and symptoms. The secondary outcomes were duration of withdrawal syndrome, duration of ICU and hospital stay, duration of intubation, development of later signs and symptoms of withdrawal syndrome, development of complications (bed sores, rhabdomyolysis, acute tubular necrosis, aspiration pneumonia, and acute respiratory distress syndrome [ARDS] due to prolonged intubation), and need for further administration of sedatives to treat agitation.

Addiction was confirmed via the history taken from the patients’ next of kin, positive urine tests, and initiation of clinical opiate withdrawal syndrome, which was determined using clinical opiate withdrawal scale (COWS). On ICU admission, simplified acute physiology score (SAPS) II was calculated for all patients. After the signs and symptoms of withdrawal initiated, the patients’ COWS was measured (4).

***2.6. Statistical analysis***

The data were recorded and transferred to statistical package for social sciences (SPSS) software version 20 and analyzed by application of Mann-Whitney U test for non-normally distributed quantitative variables and Pearson Chi-Square for categorical variables. A p value less than 0.05 was considered to be statistically significant.

## 3. Results:

A total of 48 patients (24 in each group) were included. Median age of the patients was 42 [interquartile range (IQR): 26, 56] (range: 17 to 69) years. The two groups were similar in terms of the patients’ age (p = 0.92), sex (p = 0.632), primary SAPS II score (p = 0.861), and COWS before (p = 0.537) and 120 minutes after (p = 0.136) treatment with either methadone or fentanyl (Table 1). The duration of intubation, ICU stay, and hospital stay were also similar between the two groups. The only factor that was significantly different between the two groups was the time needed for alleviation of the withdrawal signs and symptoms after the administration of the medication, which was significantly shorter in the methadone group (P=0.007). Median [IQR] clonidine administered was 0.30 [0.30, 0.60] and 0.60 [0.34, 0.60] in methadone and fentanyl groups, respectively (P = 0.16).

Eighteen patients in the methadone groups and 22 patients in the fentanyl group developed some complication during their ICU stay although this difference was statistically insignificant (p = 0.2). Seven patients (5 in methadone and two in fentanyl group) died showing a non-significant difference between the two groups (p = 0.4). Median [IQR] COWS was 18 [15, 22] (range; 3 to 27) before the administration of methadone or fentanyl, which decreased to 6 [5, 11] (range: 1 to 18) and 6 [4, 13] (range: 0 to 22) thirty and 120 minutes after administration of methadone and fentanyl, respectively (p = 0.008).

## 4. Discussion:

Our results showed that application of methadone and fentanyl controlled the withdrawal signs and symptoms with similar long-term effects, complications, hospital stay, and final outcome. The only significant difference between the groups in our study was the time needed for the medications to take effect. Patients in methadone group responded to methadone faster, although the severity of COWS was the same between the two groups after treatment. 

Opiate withdrawal occurs when opioid concentrations decrease in the central nervous system of tolerant individuals (5). The mainstay of treatment of withdrawal syndrome is replacing the opiate with an opioid with less chance of being abused. Adrenergic agonists, such as clonidine, may also be required to gain control over vital functions. General resuscitation and supportive measures are necessary for treatment in any withdrawal syndrome. A thorough history of the patient's substance use/abuse/abuse patterns should be obtained from the patient, if possible, or from the family.

Fentanyl is the preferred opioid used in many ICUs (6, 7) because of its potency and not inducing histamine release, resulting in a low risk of hemodynamic instability. However, the short duration of action of this medication often requires a continuous infusion making the weaning process more difficult (8). The use of continuous infusion sedation is associated with prolongation of mechanical ventilation and longer hospital and ICU stays (9).

Introduction of long-acting opioids via enteral administration to prevent opioid withdrawal syndrome was first described in 1965 with the use of methadone for the rehabilitation of heroin users and has been practiced in the United States since 1970 (10). 

We could not find any study in the literature that compared methadone and fentanyl in alleviation of withdrawal syndrome in dependent patients. In a similar study that compared opium tincture and methadone for controlling withdrawal syndrome in ICU-admitted dependent patients, it was concluded that a lower dose of methadone could better control the patients’ agitation (11). However, the authors claimed that the two drugs controlled the patients’ signs and symptoms fairly similar. We can claim the same because our patients showed similar results in the two groups, but received lower doses of methadone compared to the continuous infusions of fentanyl. However, it should be borne in mind that considering the equivalent doses of fentanyl and methadone, it seems that even the received doses were practically similar and methadone is only superior to fentanyl since it is administered twice a day and causes less drowsiness.

**Table 1:** Comparison of the study groups regarding the baseline characteristics and outcomes (n=56)

**Variable**	**Methadone ** **(n=28)**	**Fentanyl ** **(n=28)**	P*
Age (year)	41 (26, 55)	44 (22, 57)	0.921
SAPS II	29 (15, 36)	24 (17, 37)	0.861
COWS at the time of withdrawal	18 (15, 21)	18 (13, 22)	0.537
COWS 30 minutes after administration	6 (18, 21)	6 (4,13)	0.967
COWS 120 minutes after administration	2 (1, 3)	0 (0, 3)	0.136
Symptom after administration (minutes)	30 (30, 60)	120 (45,120)	0.008
Midazolam (mg/hour)	30 (7, 50)	12 (5, 50)	0.427
Clonidine (mg/day)	0.30 (0.30, 0.60)	0.60 (0.34, 0.60)	0.161
Duration of intubation (day)	9 (4, 17)	5 (2, 9)	0.120
Duration of ICU stay (day)	10 (7, 14)	7 (6,14)	0.572

Data are presented as median (interquartile range). *Mann-Whitney U test; SAPS: Simplified Acute Physiology Score; COWS: clinical opiate withdrawal score; ICU: intensive care unit.

## 5. Limitations

Most of our patients were methadone users. This may be a potential source of bias because it is generally considered that the best treatment for withdrawal syndrome is the same drug that has caused it. Thus, it seems that in these patients, methadone has been a better substitute from the beginning. This is a potential limitation of the current study, which should be considered and taken into account in future studies in this regard. The limited number of studied cases is another limitation. Future studies on more cases are warranted to further elucidate the superiority of methadone to fentanyl in dependent patients admitted to the ICUs.

## 6. Conclusion:

Methadone seems to treat the withdrawal signs and symptoms faster compared to fentanyl. However, these drugs seem to similarly control withdrawal signs. Further double-blinded, randomized studies with larger sample sizes are warranted to clearly determine superiority or similarity of these two medications in treatment of dependent patients in the ICUs.

## 7. Declarations:

### 7.1. Conflict of interest

None.

### 7.2. Funding:

This study was funded by Shahid Beheshti University of Medical Sciences.

### 7.3. Acknowledgment

None

### 7.4. Author contributions

All authors met the four criteria for authorship contribution based on the recommendations of the international committee of medical journal editors. SS gave the idea. BN, AM, and AH collected the data. HHM analyzed the data. NZ drafted and finalized the manuscript. 

### 7.5. Availability of data

Data is available upon request.
